# *Borrelia spielmanii* Erythema Migrans, Hungary

**DOI:** 10.3201/eid1111.050542

**Published:** 2005-11

**Authors:** Gábor Földvári, Róbert Farkas, András Lakos

**Affiliations:** * St. István University Faculty of Veterinary Science, Budapest, Hungary; † Center for Tick-borne Disease, Budapest, Hungary

**Keywords:** Borrelia spielmanii, erythema migrans, Hungary, letter

**To the Editor:** Lyme disease is the most frequent tickborne human infection in the northern hemisphere. At least 5 species of the *Borrelia burgdorferi* sensu lato complex, *B. burgdorferi* sensu stricto, *B. afzelii*, *B. garinii*, *B. bissettii*, and *B. lusitaniae*, have a pathogenic role in human Lyme disease in central Europe (*1**–**3*). A sixth pathogenic strain, A14S, has been isolated from 1 Dutch (*4*) and 2 German patients with erythema migrans (*5*). This strain was also detected in 4 questing *Ixodes ricinus* ticks in Germany (*6**,**7*) and 1 in the Czech Republic (*8*). A14S has recently been described as a new species, *B. spielmanii* (*9*); its main reservoir host is probably the garden dormouse (*Eliomys quercinus*), but *B. spielmanii* could not be detected in mice or voles. Richter et al. (*9*) could not find ticks harboring *B. spielmanii* in 3 of 5 examined areas in Germany. They were present almost exclusively in a single area where the prevalence of infection with this genotype was 15 (6%) of 251. We describe the isolation of this novel Lyme disease spirochete from a human patient with erythema migrans in Hungary.

Since 1999, we have regularly isolated *Borrelia burgdorferi* sensu lato from skin biopsy specimens of erythema migrans and acrodermatitis chronica atrophicans taken from patients at the Center for Tick-borne Diseases, Budapest, Hungary. To identify the *Borrelia* species occurring in Hungarian Lyme disease patients, we have started to molecularly analyze cultured isolates that originate from erythema migrans of different patients. DNA was isolated from 8 bacterial pellets by using QIAamp DNA mini kit (Qiagen, Hilden, Germany). Primers BSL-F and BSL-R were used; these amplify an ≈250-bp region of the outer surface protein (osp) A gene from all Lyme disease spirochetes (*10*). We added 2 μL extracted DNA to a 20-μL reaction mixture composed of 1.0 U HotStartTaq DNA polymerase, 200 μmol/L of each dNTP, 25 pmol of each primer, and 1.5 mmol/L MgCl_2_ (HotStartTaq Master Mix, Qiagen). An initial denaturation step at 94°C for 15 min was followed by 40 cycles of denaturation at 94°C for 30 s, annealing at 58°C for 30 s, and extension at 72°C for 30 s. Final extension was done at 72°C for 5 min. Amplified DNA was subjected to electrophoresis in a 1.5% agarose gel that was prestained with ethidium bromide and viewed under UV light. After purification, the dideoxy chain termination (Applied Biosystems Division, Foster City, CA, USA) was used for sequencing. Obtained sequences were checked with Chromas v.1.45 and compared to sequence data available from GenBank by using BLAST (http://www.ncbi.nlm.nih.gov/BLAST/). New sequences were submitted to GenBank.

Six sequences (DQ007298, DQ007299, DQ007300, DQ007301, DQ007302, DQ007303) showed 100% homology to *B. afzelii* Khab 625 strain (AY502599). One (DQ007297) of the remaining 2 samples showed 99.6% similarity with *B. burgdorferi* B31 (AE000790), and the other (AY995900) showed 99.21% similarity with *B. spielmanii* (AF102057).

The patient whose culture showed *B. spielmanii* was a 42-year-old woman with a homogenous erythema migrans, diagnosed on September 24, 1999. The erythema was 10 cm in diameter on the front surface of the knee at the first visit (Figure). The immunoglobulin M (IgM) and IgG *Borrelia* immunoblot that applied *B. afzelii* (ACA1) antigen was negative in serum drawn on the seventh day after the appearance of erythema migrans. The patient did not remember a tick bite and had not traveled abroad during the previous 6 months. She complained of an "extremely unusual," intense, serous nasal discharge that started 3 weeks before the appearance of erythema migrans and of a moderate headache; both disappeared spontaneously 2 weeks before treatment.

**Figure:**
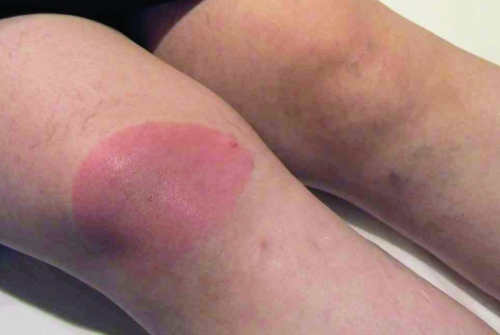
Erythema migrans caused by Borrelia spielmanii.

Our results show at least 3 distinct species of *B. burgdorferi* sensu lato in Hungary. In addition to *B. burgdorferi* sensu stricto and *B. afzelii*, known throughout Europe, we detected the recently described species *B. spielmanii* among randomly selected samples. Together with 2 previous publications (*4**,**5*), our observation also suggest that *B. spielmanii* has a pathogenic role in human Lyme disease. Although *B. spielmanii* is distributed more focally than other species of the *B. burgdorferi* sensu lato complex (*9*), it occurs from the Netherlands through Germany and Czech Republic to Hungary (*4**,**5**,**7**,**8*).
